# Effectiveness of Variable-Gain Kalman Filter Based on Angle Error Calculated from Acceleration Signals in Lower Limb Angle Measurement with Inertial Sensors

**DOI:** 10.1155/2013/398042

**Published:** 2013-10-27

**Authors:** Yuta Teruyama, Takashi Watanabe

**Affiliations:** Graduate School of Biomedical Engineering, Tohoku University, Sendai 980-8579, Japan

## Abstract

The wearable sensor system developed by our group, which measured lower limb angles using Kalman-filtering-based method, was suggested to be useful in evaluation of gait function for rehabilitation support. However, it was expected to reduce variations of measurement errors. In this paper, a variable-Kalman-gain method based on angle error that was calculated from acceleration signals was proposed to improve measurement accuracy. The proposed method was tested comparing to fixed-gain Kalman filter and a variable-Kalman-gain method that was based on acceleration magnitude used in previous studies. First, in angle measurement in treadmill walking, the proposed method measured lower limb angles with the highest measurement accuracy and improved significantly foot inclination angle measurement, while it improved slightly shank and thigh inclination angles. The variable-gain method based on acceleration magnitude was not effective for our Kalman filter system. Then, in angle measurement of a rigid body model, it was shown that the proposed method had measurement accuracy similar to or higher than results seen in other studies that used markers of camera-based motion measurement system fixing on a rigid plate together with a sensor or on the sensor directly. The proposed method was found to be effective in angle measurement with inertial sensors.

## 1. Introduction

Wearable inertial sensors have been used in many studies to estimate human kinetic data. Those sensors have advantages of low cost, small size, and practical usefulness compared to traditional lab tools such as optical motion measurement system or electric goniometers. As has been reported in previous studies, segment and joint angles [1–11], stride length [1, 3, 5], walking speed [1, 5], gait event timing [5, 12, 13], and so on can be estimated using inertial sensors. Therefore, a wearable inertial sensor system can be effective for objective and quantitative evaluation in rehabilitation of motor function. That is, inertial sensors are considered to be suitable for clinical applications.

In our previous studies, a method of measuring lower limb angles using wireless inertial sensors was developed to realize simplified wearable gait evaluation system for rehabilitation support. The method was tested in measurement of gait of healthy subjects [14, 15]. Although the method was shown to have practical accuracy, measurement errors varied depending on movement speeds or subjects. In the angle measurement method of our previous studies, Kalman filter was applied using angle calculated from acceleration signals. Many other studies also used accelerometers as inclinometers to measure inclination angles of body segments [2, 5, 7, 8]. However, the angle calculated from acceleration signals was influenced by impact and movement accelerations, since the angle was calculated from gravitational acceleration. Therefore, those impact and movement accelerations can be considered as one of the causes of variation of measurement error.

Low-pass filtering of acceleration signals is one of the methods to reduce influences of those impact and motion accelerations. In our previous studies, outputs of accelerometer were filtered with Butterworth low-pass filter with cutoff frequency of 0.5 Hz [14, 15]. However, low cutoff frequency is at risk for increasing measurement error because of its large time constant in the low-pass filtering. Therefore, using higher cutoff frequency has a possibility to improve measurement error.

In this paper, in order to reduce influences of impact and movement accelerations in calculation of angles using Kalman filter, a variable-Kalman-gain method with higher cutoff frequency for the low-pass filtering was tested. In previous studies, a method to change Kalman gain based on the magnitude of acceleration signals was used [9, 16]. However, in this study, the method to change Kalman gain based on error of angle calculated from acceleration signals was proposed. This is because the Kalman filter of our system is applied using the angles calculated from acceleration signals.

The variable-gain method was evaluated in measurement of lower limb angles of healthy subjects in treadmill walking using a camera-based motion measurement system to measure reference angles for evaluation. The fixed-Kalman-gain method and the variable-gain method based on magnitude of acceleration signals were compared to the proposed method. Then, similar evaluation was performed in measurement of angles of a rigid body model, because some other studies evaluated angle measurement method with inertial sensors using a rigid plate that fixed a sensor together with markers of an optical motion measurement system or attaching the markers directly on the sensor [4, 8, 10, 11].

## 2. Angle Measurement Method Based on Kalman Filter

### 2.1. Fixed-Kalman-Gain Method

**Figure 1:**
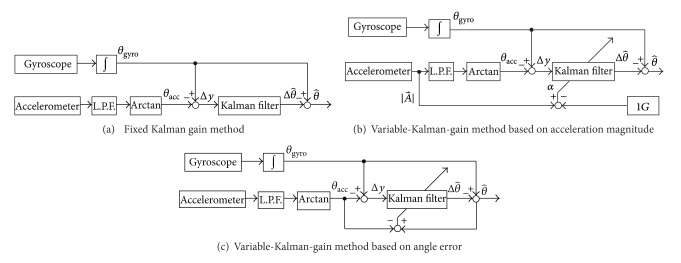
Block diagrams of the angle calculation methods using Kalman filter tested in this paper.

Figure 1(a) shows the block diagram of the angle measurement method used in our previous studies. An inclination angle of body segment is calculated by integrating an output of a gyroscope. Here, the integration error is corrected by Kalman filter using the angle calculated from outputs of an accelerometer. Then, joint angle is calculated from difference of inclination angles of the adjacent segments.

The state equation is represented by error of angle measured with a gyroscope Δ*θ* and bias offset of outputs of the gyroscope Δ*b* as follows:

(1) $$\begin{array}{c} \left[\begin{array}{c} \Delta \theta_{k+1}\\ \Delta b_{k+1} \end{array}\right]=\left[\begin{array}{cc} 1 & \Delta t\\ 0 & 1 \end{array}\right]\left[\begin{array}{c} \Delta \theta_{k}\\ \Delta b_{k} \end{array}\right]+\left[\begin{array}{c} \Delta t\\ 1 \end{array}\right]w, \end{array}$$

where *w* is error in measurement with the gyroscope and Δ*t* is sampling period. Observation signal is deference of angles obtained from the gyroscope and an accelerometer Δ*y*, which is given by

(2) $$\begin{array}{c} \Delta y_{k}=\left[\begin{array}{cc} 1 & 0 \end{array}\right]\left[\begin{array}{c} \Delta \theta_{k}\\ \Delta b_{k} \end{array}\right]+v, \end{array}$$

where *v* is error in measurement with the accelerometer. On this state-space model, Kalman filter repeats corrections (3) and predictions (4):

(3) $$\begin{array}{c} \left[\begin{array}{c} \Delta {\hat{\theta }}_{k}\\ \Delta {\hat{b}}_{k} \end{array}\right]=\left[\begin{array}{c} \Delta {\hat{\theta }}_{k}^{-}\\ \Delta {\hat{b}}_{k}^{-} \end{array}\right]+\left[\begin{array}{c} K_{1}\\ K_{2} \end{array}\right]\left(\Delta y_{k}-\Delta {\hat{\theta }}_{k}^{-}\right), \end{array}$$

(4) $$\begin{array}{c} \left[\begin{array}{c} \Delta {\hat{\theta }}_{k+1}^{-}\\ \Delta {\hat{b}}_{k+1}^{-} \end{array}\right]=\left[\begin{array}{cc} 1 & \Delta t\\ 0 & 1 \end{array}\right]\left[\begin{array}{c} \Delta {\hat{\theta }}_{k}\\ \Delta {\hat{b}}_{k} \end{array}\right], \end{array}$$

where *K*_1_ and *K*_2_ represent Kalman gain for Δ*θ* and Δ*b*, respectively. Notations such as $\Delta \hat{\theta }$ and $\Delta {\hat{\theta }}_{}^{-}$ represent estimated value and predicted value for Δ*θ*, respectively. For the initial condition, $\Delta {\hat{\theta }}_{0}^{-}$ was set as 0, and $\Delta {\hat{b}}_{0}^{-}$ was set as $\Delta \hat{b}$ at the last measurement. The Kalman filter was applied repeatedly until its output converged.

Values of Kalman gain were fixed in angle calculation in our previous studies. Those gain values are determined by the noise ratio, that is, the ratio of the covariance of observation noise and covariance of process noise. In our system, value of Kalman gain increases as the noise ratio decreases and decreases as the noise ratio increases.

As shown in (3) and (4), the Kalman filter estimates Δ*θ* and Δ*b* by using the angle difference Δ*y*. Therefore, large value of Kalman gain (small noise ratio) means that calculation results become highly dependent on accelerometer, while small Kalman gain (large noise ratio) means that calculation results become highly dependent on gyroscope. Considering power of the correction by Kalman filter, values of the noise ratio were determined by trial and error method.

### 2.2. Variable-Kalman-Gain Method

The fixed-Kalman-gain method shown in the previous section was found to be useful in measurement of angles during gait of healthy subjects [14, 15]. However, impact and movement accelerations were considered to increase measurement error and its variation. That is, those accelerations are considered to cause inappropriate correction by the Kalman filter. As described in the previous section, Kalman gain means correction power of the Kalman filter. Therefore, in this paper, following two methods of changing the noise ratio to determine Kalman gain were tested.

(a) *Acceleration Magnitude-Based Method*.  Figure 1(b) shows the variable-gain method based on acceleration magnitude, which was introduced in reference to previous studies [9, 16]. Value of the noise ratio is adjusted based on the magnitude of impact and motion acceleration signals. Here, the magnitude of impact and motion acceleration signals was calculated by subtracting gravitational acceleration (1 G) from magnitude of measured acceleration vector. That is, the value of noise ratio is varied as follows:

(5) $$\begin{array}{c} n=n_{1},\;\text{for}\;\left|\alpha \right|\leq \alpha_{1},\\ n=n_{2},\;\text{for}\;\alpha_{1}<\left|\alpha \right|\leq \alpha_{2},\\ n=n_{3},\;\text{for}\;\alpha_{2}<\left|\alpha \right|\leq \alpha_{3},\\ n=n_{4},\;\text{for}\;\alpha_{3}<\left|\alpha \right|, \end{array}$$

where *n* and |*α*| represent the noise ratio and the magnitude of impact and motion acceleration signals, respectively. *α*_1_, *α*_2_, and *α*_3_ show thresholds to change the value of noise ratio, respectively.

(b) *Angle Error-Based Method.* Figure 1(c) shows the method proposed in this paper, in which the noise ratio is adjusted based on the difference between the angle estimated by the Kalman filter and the angle calculated from acceleration signals. Here, the angle difference was used approximately as the magnitude of influence of impact and motion accelerations. That is, it was assumed that the angle difference involves substantial error of angle calculated from acceleration signals, which is caused by impact and movement accelerations. The value of noise ratio is varied as follows:

(6) $$\begin{array}{c} n=n_{1},\;\text{for}\;\left|\hat{\theta }-\theta_{\text{acc}}\right|\leq \theta_{1},\\ n\;=\;n_{2},\;\text{for}\;\theta_{1}<\left|\hat{\theta }-\theta_{\text{acc}}\right|\leq \theta_{2},\\ n=n_{3},\;\text{for}\;\theta_{2}<\left|\hat{\theta }-\theta_{\text{acc}}\right|\leq \theta_{3},\\ n=n_{4},\;\text{for}\;\theta_{3}<\left|\hat{\theta }-\theta_{\text{acc}}\right|, \end{array}$$

where, *n* and $\left|\hat{\theta }-\theta_{\text{acc}}\right|$ represent the noise ratio and the angle difference between the angle estimated by the Kalman filter $\hat{\theta }$ and the angle calculated from acceleration signals *θ*_acc_, respectively. The angle difference $\left|\hat{\theta }-\theta_{\text{acc}}\right|$ shows approximately the magnitude of influence of impact and motion accelerations. *θ*_1_, *θ*_2_, and *θ*_3_ show thresholds to change values of the noise ratio, respectively.

## 3. Methods of Validation Tests

The angle calculation methods were applied to data measured with inertial sensors and evaluated in comparison to those angles measured with an optical motion measurement system. First, the evaluation was performed in measurement of lower limb angles in treadmill walking with healthy subjects. Then, angles of a rigid body model were measured for evaluation of the methods, because some other studies evaluated their angle measurement method with inertial sensors using the rigid plate that fixed a sensor together with markers of an optical motion measurement system [4, 8, 10, 11].

### 3.1. Measurement of Lower Limb Angles in Treadmill Walking

Inclination angles of lower limb segments in treadmill walking were measured with 3 healthy subjects (male, 22-23 y.o.). The subjects walked on a treadmill for about 90 sec at speeds of 1 km/h (slow), 3 km/h (normal), and 5 km/h (fast). Five trials were performed for each walking speed.

**Figure 2:**
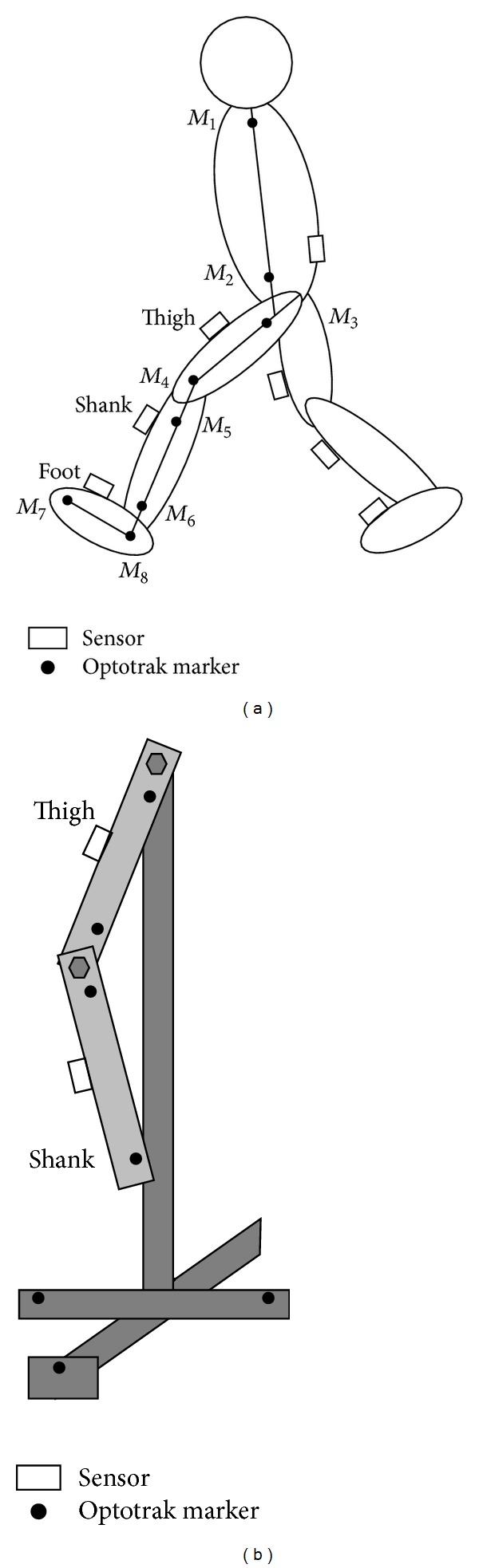
Experimental setup for the angle measurement during treadmill walking (a) and the angle measurement using rigid body model (b). M1: the acromion, M2: along the long axis of the trunk at the same height as the iliospinale anterius, M3: the great trochanter, M4: the lateral femoral condyle, M5: the caput fibulae, M6: the lateral malleolus, M7: the metatarsale fibulare, and M8: on the foot at the same height as the metatarsale fibulare along the line of shank markers.

Seven wireless inertial sensors (WAA-006, Wireless Technologies) were attached on the feet, the shanks and the thighs of both legs, and lumbar region with stretchable bands (Figure 2(a)). The sensors were put inside of pocket of the band. Acceleration and angular velocity signals of each sensor were measured with a sampling frequency of 100 Hz and were transmitted to a PC via Bluetooth network.

The optical motion measurement system (Optotrak, Northern Digital, Inc.) was used to measure reference data for evaluating angles calculated by the methods from data measured with the inertial sensors. Markers for reference data were attached on the left side of the body (Figure 2(a)). The maker positions were measured with a sampling frequency of 100 Hz.

### 3.2. Measurement of Angles with a Rigid Body Model

Figure 2(b) shows the schematic diagram of the rigid body model used in the measurement. The rigid body model simulated motion of the thigh, the shank, and the knee joint. The optical motion measurement system (Optotrak, Northern Digital, Inc.) was also used to measure reference data for evaluating the angle calculation method. Sensors and markers were attached on the rigid body model as shown in Figure 2(b). Acceleration and angular velocity signals of each sensor and the marker positions were measured with sampling frequency of 100 Hz.

Inclination angles of the thigh and the shank parts were measured for 35 sec with angle ranges of ±15, ±30, ±45, ±60, and ±75 deg for the thigh part. Zero degree means the direction of gravitational force. The shank part was moved freely associated with movement of the thigh part. The cycle period of the movements was 2 sec, and five trials were conducted for each target angle range.

## 4. Results of Validation Tests

Two variable-Kalman-gain methods were evaluated in comparison to the previous fixed-Kalman-gain method. Here, for the fixed-gain method, values of the noise ratio *n* to determine Kalman gain and the cutoff frequency of Butterworth low-pass filter for acceleration signals *f*_*c*_ are shown below.

 Method 1 (previous fixed Kalman gain method)
  (7) $$\begin{array}{c} n\;=10^{6},\;\;f_{c}=\;0.5\;\text{Hz}. \end{array}$$
 Method 2 (fixed Kalman gain method)
  (8) $$\begin{array}{c} n=10^{6},\;\;f_{c}=10\;\text{Hz}. \end{array}$$

Method 1 is the previous method used in our research group, in which the noise ratio of Kalman filter was fixed and cutoff frequency of the low-pass filter for acceleration signals was determined to remove impact and motion accelerations. Method 2 is the fixed-Kalman-gain method with higher cutoff frequency of the low-pass filter, which was tested to make clear the influence of low cutoff frequency on measurement error.

There was offset difference between the sensor system and camera-based motion analysis system, because the markers for the reference signals were not attached on the sensors. Therefore, the difference was calculated as the mean value of the first 100 samples of the 1st measurement and removed the value for evaluation. Then, root mean squared error (RMSE) and correlation coefficient (*ρ*) between measured angles with sensors and reference values were calculated for evaluating measurement accuracy. In this paper, inclination angles of lower limb segments in the sagittal plane were evaluated.

### 4.1. Measurement of Lower Limb Angles in Treadmill Walking

For the variable-Kalman-gain methods, values of the noise ratio *n* and threshold values were determined by trial and error as shown below.

 Method 3 (variable-gain method based on acceleration magnitude)
  (9) $$\begin{array}{c} n=10^{4},\;\text{for}\;\left|\alpha \right|\leq 20\;\text{mG},\\ n=10^{6},\;\text{for}\;20\;\text{mG}<\left|\alpha \right|\leq 300\;\text{mG},\\ n=10^{8},\;\text{for}\;300\;\text{mG}<\left|\alpha \right|\leq 1\;\text{G},\\ n=10^{13},\;\text{for}\;1\;\text{G}<\left|\alpha \right|. \end{array}$$
 Method 4 (variable-gain method based on angle error)
  (10) $$\begin{array}{c} n=10^{4},\;\text{for}\;\left|\hat{\theta }-\theta_{\text{acc}}\right|\leq 1\;\deg,\\ n\;=\;10^{6},\;\text{for}\;1\;\deg<\left|\hat{\theta }-\theta_{\text{acc}}\right|\leq 15\;\deg,\\ n=10^{8},\;\text{for}\;15\;\deg<\left|\hat{\theta }-\theta_{\text{acc}}\right|\leq 60\;\deg,\\ n=10^{13},\;\text{for}\;60\;\deg<\left|\hat{\theta }-\theta_{\text{acc}}\right|. \end{array}$$

Here, the cutoff frequency of the low-pass filter for acceleration signals *f*_*c*_ was 10 Hz for both methods.

**Figure 3:**
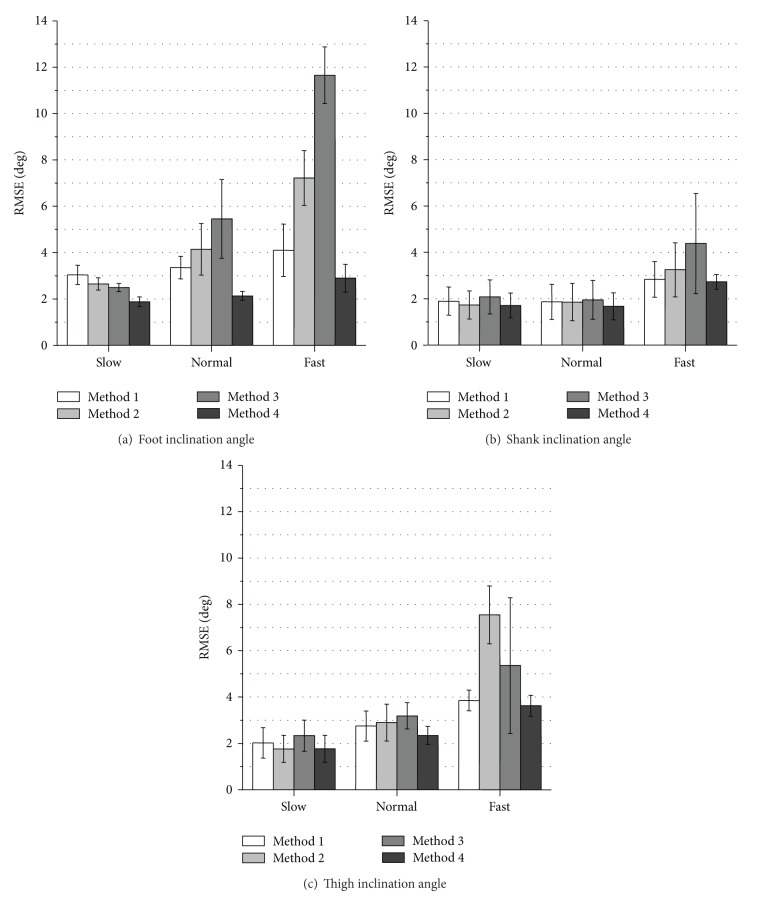
Evaluation results of RMSE of measured inclination angles during treadmill walking. Average values obtained from the results of 5 trials of all subjects are shown for each walking speed.

**Figure 4:**
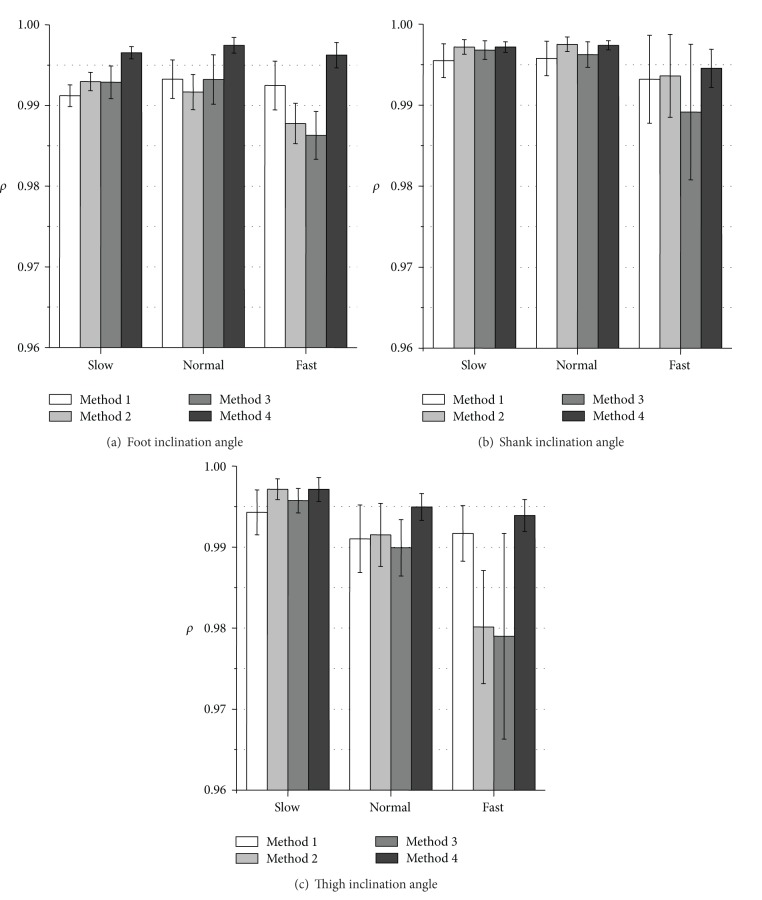
Evaluation results of correlation coefficient (*ρ*) of measured inclination angles during treadmill walking. Average values obtained from the results of 5 trials of all subjects are shown for each walking speed.

Figures 3 and 4 show RMSE values and *ρ* values of measured inclination angles, respectively. The proposed variable-gain method (Method 4) showed the smallest average RMSE values and the largest *ρ* values for all of measurement conditions. Method 4 achieved average RMSE values of less than 3.0 deg except for thigh angle at fast walking speed and average values of correlation coefficient larger than 0.994 for all the measurement conditions.

The measurement accuracy of foot inclination angle was improved significantly with Method 4 for all walking speeds comparing to the results of Method 1 used in our previous studies (Figures 3(a) and 4(a)). For the shank and the thigh inclination angles, slight improvement of RMSE values and *ρ* values was shown for all walking speeds with Method 4 compared to the results of Method 1 (Figures 3(b), 3(c) and 4(b), 4(c)).

The values of RMSE with Method 2 (fixed-gain method with higher cut-off frequency) decreased compared to the results of Method 1 in all the segments at slow walking speed. However, at normal and fast walking speeds, the values of RMSE with Method 2 increased compared to the results of Method 1. Method 3 (variable-gain method based on acceleration magnitude) reduced measurement accuracy especially for fast waking speed and for foot inclination angle.

### 4.2. Measurement of Angles Using Rigid Body Model

The parameter values used for Methods 3 and 4 are shown below.

 Method 3 (variable-gain method based on acceleration magnitude)
  (11) $$\begin{array}{c} n=10^{4},\;\text{for}\;\left|\alpha \right|\leq 10\;\text{mG},\\ n=3\;\times \;10^{6},\;\text{for}\;10\;\text{mG}<\left|\alpha \right|\leq 200\;\text{mG},\\ n=\;10^{7},\;\text{for}\;200\;\text{mG}<\left|\alpha \right|\leq 400\;\text{mG},\\ n=2\;\times \;10^{7},\;\text{for}\;400\;\text{mG}<\left|\alpha \right|. \end{array}$$
 Method 4 (variable-gain method based on angle error)
  (12) $$\begin{array}{c} n=10^{4},\;\text{for}\;\left|\hat{\theta }-\theta_{\text{acc}}\right|\leq 1\;\deg,\\ n\;=3\times 10^{6},\;\text{for}\;1\;\deg<\left|\hat{\theta }-\theta_{\text{acc}}\right|\leq 20\;\deg,\\ n=10^{7},\;\text{for}\;20\;\deg<\left|\hat{\theta }-\theta_{\text{acc}}\right|\leq 30\;\deg,\\ n=2\times 10^{7},\;\text{for}\;30\;\deg<\left|\hat{\theta }-\theta_{\text{acc}}\right|. \end{array}$$

Here, the cutoff frequency of the low-pass filter for acceleration signals *f*_*c*_ was 10 Hz for both methods. The parameter values of Methods 3 and 4 were changed from those values in angle measurement with human subjects, since magnitude of impact and motion acceleration signals and angle difference $\left|\hat{\theta }-\theta_{\text{acc}}\right|$ were smaller than that in the measurement with human subjects.

**Figure 5:**
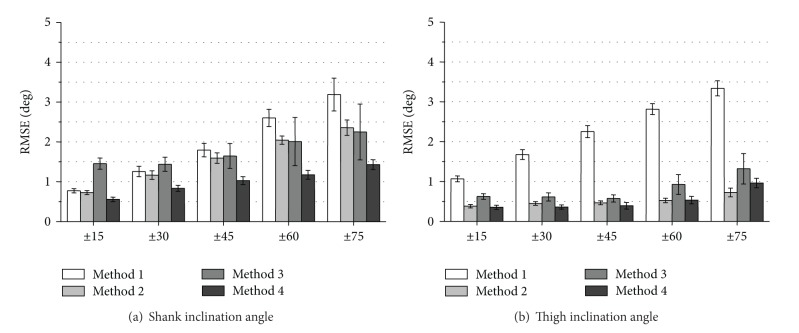
Evaluation results of RMSE of measured inclination angles using rigid body model. Average values obtained from the results of 5 trials are shown for each target angle range.

**Figure 6:**
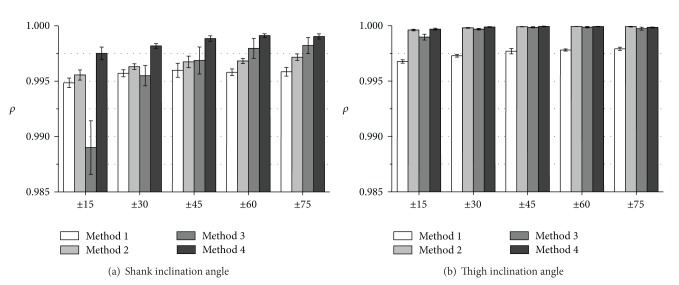
Evaluation results of correlation coefficient (*ρ*) of measured inclination angles using rigid body model. Average values obtained from the results of 5 trials are shown for each target angle range.

Figures 5 and 6 show RMSE values and *ρ* values of measured inclination angles, respectively. The proposed variable-gain method (Method 4) showed highest measurement accuracy almost for all the target angle ranges. Average values of RMSE and correlation coefficient with Method 4 were less than 1.5 deg⁡ and larger than 0.9975, respectively. Although Methods 2 and 3 were also effective to improve measurement accuracy, Method 3 could not improve shank angle in movements of target angle range of ±15 deg and ±30 deg.

## 5. Discussion

The proposed variable-Kalman-gain method (Method 4) measured lower limb angles in treadmill walking with the smallest average values of RMSE and the largest average values of correlation coefficient. In particular, Method 4 showed significant improvement in calculation of foot inclination angle in treadmill walking compared to our previous method (Method 1). It is a useful result that measurement accuracy of foot inclination angle was improved, because evaluation of foot movements in walking is important for gait of motor disabled subjects and elderly persons. In measurement of shank and thigh angles, values of the noise ratio were not so greatly varied, since magnitude of the angle difference $\left|\hat{\theta }-\theta_{\text{acc}}\right|$ did not fluctuate significantly during movements. This is one of the reasons why improvement of measurement accuracy was not so large in shank and thigh angles with Method 4.

**Figure 7:**
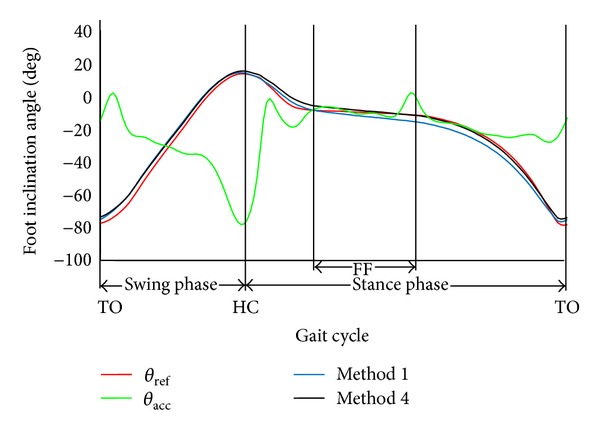
An example of waveforms of reference and measured foot inclination angles at normal walking speed. *θ*_ref_ and *θ*_acc_ represent reference angle and angle calculated from acceleration signals, respectively. The *x*-axis shows the gait cycle. The toe off, the heel contact, and the foot flat are represented by TO, HC, and FF, respectively.

The proposed variable-Kalman-gain method (Method 4) was highly effective in calculation of foot inclination angle.  Figure 7 shows the reference and calculated foot inclination angles of one gait cycle at normal walking speed. As seen in Figure 7, the calculated angle with Method 4 was almost equal to that with Method 1 between around the TO and the HC in the swing phase. Method 4 improved angle calculation between around the FF and the TO in the stance phase. It is considered that the sensor attached on the foot was close to the stationary state at around the FF. At that time, the variable-Kalman-gain method corrected the angle significantly increasing values of Kalman gain (decreasing noise ratio), since the angle difference $\left|\hat{\theta }-\theta_{\text{acc}}\right|$ was small as the influence of impact and movement accelerations was small. It is possible to decrease angle measurement error between the FF and the TO by reducing noise ratio with Method 1. However, in that case, angle error between around the TO and the HC is increased by the influence of impact and movement accelerations. Method 4 reduced Kalman gain effectively at around the TO and the HC, since the angle difference $\left|\hat{\theta }-\theta_{\text{acc}}\right|$ increased. Therefore, the Method 4 could be effective especially in foot angle measurement, decreasing the influence of impact and motion accelerations.

As shown in Figure 3, RMSE values of measured inclination angles in treadmill walking with Method 2 decreased at slow walking speed compared to the results of Method 1 in all segments. In addition, as shown in Figure 5, RMSE values of measured inclination angles of the rigid body model with Method 2 decreased at all target angle ranges compared to the results of Method 1 for both segments. These suggest that very low cutoff frequency for the low-pass filtering of the acceleration signal increases measurement error if impact and movement accelerations are not so large. The cause of error increases in treadmill walking at the normal and fast walking speeds with Method 2 is considered to be influences of impact and motion accelerations. It is considered that although 0.5 Hz of cutoff frequency is reasonable value for removing impact and motion accelerations in angle calculation of human gait, error increase is caused by the large delay in the low pass filtering.

The variable-gain method based on acceleration magnitude (Method 3) did not show improvement of measurement accuracy of lower limb angles of human gait. For most of measurement conditions, average values of RMSE increased, and those of correlation coefficient decreased. The parameter values that are good for changing Kalman gain used in (9)–(12) were determined by trial and error method for both variable-gain methods. Although there is a possibility of improving measurement accuracy with Methods 3 and 4, the results of this paper suggest that the proposed method of changing the Kalman gain based on angle error was more suitable to the Kalman filter used in our system than that based on acceleration magnitude.

In angle measurement of the rigid body model, the values of RMSE with Method 4 were less than 1.5 deg for all segments and all target angle ranges. Those RMSE values show measurement accuracy similar to or higher than results seen in other studies that used markers of camera-based motion measurement system fixing on a rigid plate together with the sensor or on the sensor directly [4, 8, 10, 11]. It is considered that the proposed variable-gain method became effective in measurement of human gait.

**Figure 8:**
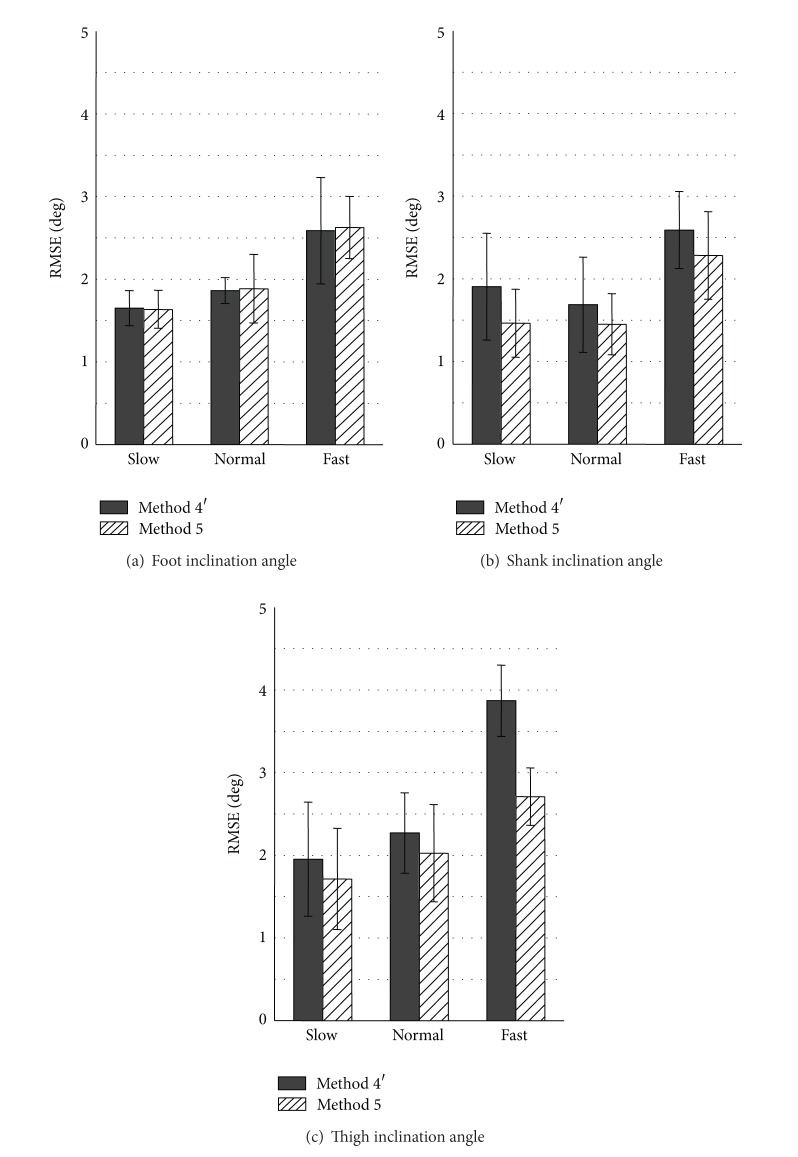
Average values of RMSE of measured inclination angles during treadmill walking calculated by using Methods 4 and 5.

In the proposed variable-gain method, influence of impact and motion accelerations was approximately represented by $\left|\hat{\theta }-\theta_{\text{acc}}\right|$. Here, the approximation was validated by comparing to results of using |*θ*_ref_ − *θ*_acc_| as shown in Figure 8, in which *θ*_ref_ shows reference value measured with the camera-based motion measurement system. In the comparison, the noise ratio (Kalman gain) was determined as continuous value by the followings from the angle difference.

Method 4′:

(13) $$\begin{array}{c} n=10^{4}e^{0.46\cdot |\hat{\theta }-\theta_{\text{acc}}|}. \end{array}$$

Method 5:

(14) $$\begin{array}{c} n=10^{4}e^{0.46\cdot |\theta_{\text{ref}}-\theta_{\text{acc}}|}. \end{array}$$

Here, (13) and (14) were derived by linear approximation of discrete values of the noise ratio *n* in (10). That is,

(15) $$\begin{array}{c} \log n=ax+\log b, \end{array}$$

where *a* and *b* represent constant values and *x* represents the angle difference. The constant value *a* was determined to decrease RMSE values by trial and error method, and *b* was set 10^4^ from (10). As shown in Figure 8, for foot inclination angles, measurement results with Method 5 were similar to the results with Method 4′ for all walking speeds. Measurement results for the shank and the thigh inclination angles were improved for all walking speeds with Method 5 using reference values. Average values of RMSE with Method 5 were less than 3.0 deg for all walking speeds and all segments. This result suggests that the proposed variable-Kalman-gain method based on angle error is effective to improve measurement accuracy of angle during human gait. It is also suggested that measurement accuracy with Method 4′ can be improved if error in $\left|\hat{\theta }-\theta_{\text{acc}}\right|$ is reduced.

**Figure 9:**
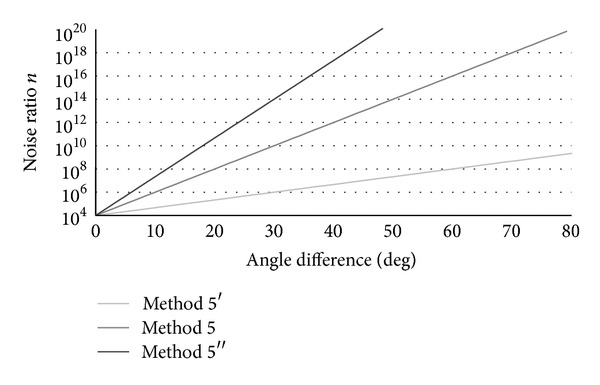
Relationships between the noise ratio and the angle difference used in the test of influence of the noise ratio.

**Figure 10:**
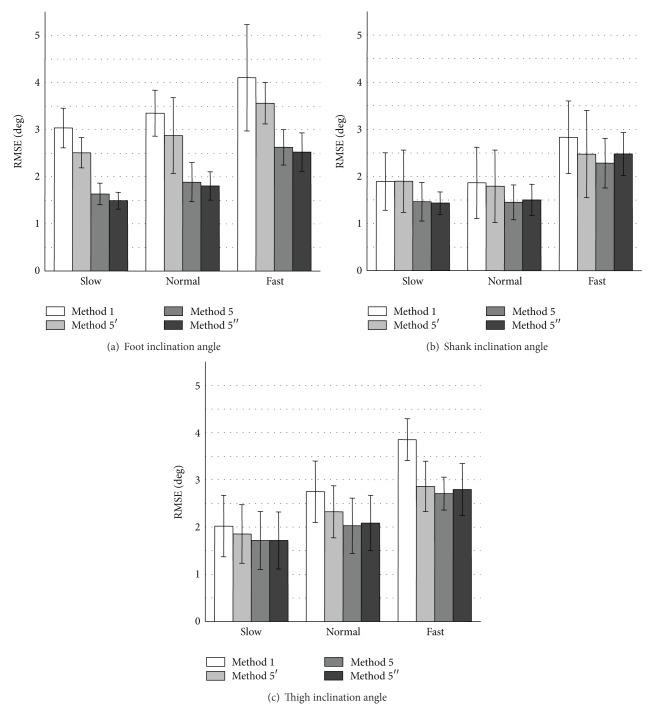
Average RMSE values of measured inclination angles during treadmill walking calculated by fixed-gain method (Method 1) and variable-gain method with 3 different parameter settings for the noise ratio.

Angle measurement accuracy depends on the noise ratio. In this paper, parameter values to calculate values of noise ratio used in (9)–(14) were determined to decrease RMSE values by trial and error method. Here, different relationships between the noise ratio and the angle difference described by (14) were examined. Figures 9 and 10 show the tested relationships and results of their measurement accuracy, respectively. Method 5′ used smaller noise ratio and Method 5′′ used larger noise ratio than Method 5. In Figure 10, the results obtained by Method 1 (fixed-gain method) are also shown. The RMSE values were decreased with variable-gain method in any parameter setting compared to the fixed-gain method. However, Method 5′ (smaller noise ratio) showed larger RMSE values than Method 5 for all the measurement conditions. It is considered that the influence of impact and movement accelerations was not decreased sufficiently with Method 5′ because of large value of Kalman gain (small noise ratio). On the other hand, RMSE values with Method 5′′ (larger noise ratio) were similar to that of Method 5 for almost all the measurement conditions. However, Method 5′′ has a tendency to increase RMSE values of the thigh and the shank inclination angles for the fast walking speed. Therefore, further studies on the method to determine appropriate Kalman gain are expected. In addition, this paper focused only on the error of angle calculated from acceleration signals in determination of Kalman gain. It is considered that the noise ratio also depends on magnitude of the offset drift of gyroscope. This is also required to be studied more for measurement of angles with the variable-gain Kalman filter. Finally, the proposed variable-Kalman-gain method was validated in measurement of inclination angles of lower limb segments in the sagittal plane in this paper. It is expected to show the effectiveness of the proposed method in measurement of 3 dimensional angles.

## 6. Conclusion

In this paper, variable-gain Kalman filter was tested to improve measurement accuracy of lower limb angles during gait, in which two calculation methods of Kalman gain were compared to fixed-gain Kalman filter. In measurements of lower limb angles of healthy subjects in treadmill walking and that of angles of a rigid body model, the variable-gain method based on the angle difference proposed in this study showed the highest measurement accuracy for most of measurement conditions. In particular, the proposed variable-gain method improved significantly measurement accuracy of foot inclination angle in human gait. On the other hand, measurement results of the shank and the thigh inclination angles show slight improvement of measurement accuracy. The proposed variable-gain method was found to be effective in angle measurement with inertial sensors. The results also suggested that more accurate measurement can be realized by improving estimation accuracy of the angle difference |*θ*_ref_ − *θ*_acc_|. Further studies on this point and to find appropriate method to determine Kalman gain using the angle difference and other parameters are expected.
